# Simplified definition and imaging-based stratification of at-risk MASH using magnetic resonance elastography

**DOI:** 10.1097/HC9.0000000000001024

**Published:** 2026-08-11

**Authors:** Jiahui Li, Hao Wu, Kevin J. Glaser, Fang-Shu Ou, Armando Manduca, Sudhakar K. Venkatesh, Claude Sirlin, Rohit Loomba, Vijay H. Shah, Richard L. Ehman, Alina M. Allen, Meng Yin

**Affiliations:** 1 Department of Radiology, Mayo Clinic, Rochester, Minnesota, USA; 2 Division of Clinical Trials and Biostatistics, Department of Quantitative Health Sciences, Mayo Clinic, Rochester, Minnesota, USA; 3 Department of Radiology, University of California at San Diego, La Jolla, California, USA; 4 Division of Gastroenterology and Hepatology, Department of Medicine, University of California at San Diego, La Jolla, California, USA; 5 Division of Gastroenterology and Hepatology, Department of Medicine, Mayo Clinic, Rochester, Minnesota, USA

**Keywords:** fibrosis, NASH, noninvasive, resmetirom, semaglutide

## Abstract

**Background:**

Accurate identification of patients with metabolic dysfunction–associated steatohepatitis (MASH) at risk of disease progression (at-risk MASH) is critical for guiding resmetirom and semaglutide therapy. Inconsistent definitions of at-risk MASH complicate clinical risk classification and comparisons across studies. This study aimed to standardize the definition of at-risk MASH and propose optimized MRE-based liver stiffness (LS-MRE) cutoffs to improve noninvasive risk stratification and better guide pharmacotherapy.

**Methods:**

In this prospective study, 413 patients with suspected or diagnosed MASLD from two medical centers underwent liver biopsy and MRE.

**Results:**

A simplified definition of at-risk MASH (MASH with fibrosis stage≥2) identified a broader group of patients requiring clinical attention than a stricter definition requiring NAFLD Activity Score≥4 with ≥1 point in steatosis, inflammation, and ballooning (n=122 vs. 77). LS-MRE alone showed the highest diagnostic accuracy for identifying at-risk MASH using the simplified definition (AUC 0.91 [0.87, 0.95]) and cirrhosis (AUC 0.93 [0.88, 0.99]), compared with FIB-4 and individual laboratory parameters (AST, ALT, and platelet count) (*p*<0.01 for all, paired DeLong tests). Optimized LS-MRE cutoffs (2.8–6.4 kPa) captured 25 additional biopsy-proven pharmacotherapy candidates (28% of all eligible patients) compared to AASLD guidelines–recommended cutoffs (3.1–4.4 kPa), improving sensitivity (80% vs. 50%) with acceptable specificity (73% vs. 88%).

**Conclusions:**

A simplified definition of at-risk MASH enables broader identification of treatment candidates. LS-MRE demonstrates excellent diagnostic performance and improves noninvasive risk stratification when paired with optimized cutoffs. These findings support the use of MRE to guide resmetirom and semaglutide eligibility and improve access to emerging therapies.

## Graphical abstract

**Figure:**
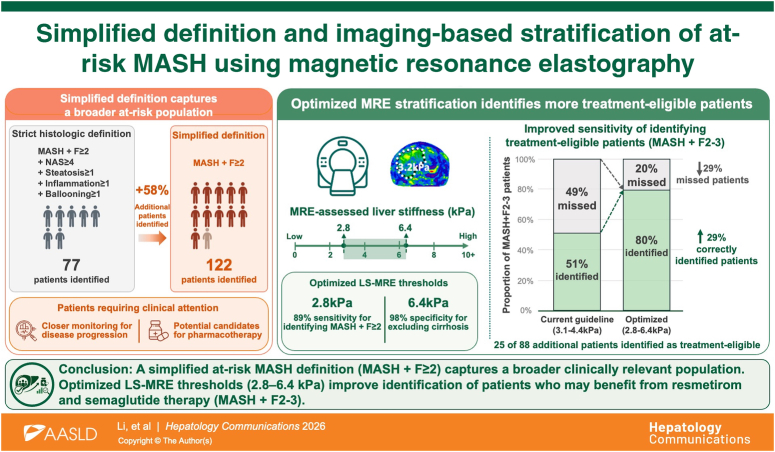

## INTRODUCTION

Metabolic dysfunction–associated steatotic liver disease (MASLD), formerly known as NAFLD, is a leading cause of chronic liver disease, affecting approximately one-third of the global population.^1^,^2^,^3^ Among individuals with MASLD, about 16% progress to metabolic dysfunction–associated steatohepatitis (MASH), previously known as NASH,^4^ which is histologically characterized by hepatic steatosis, ballooning, and lobular inflammation. As the progressive form of the disease, MASH carries a higher risk of advancing to cirrhosis and eventually HCC.^5^,^6^ With the Food and Drug Administration approval of resmetirom and semaglutide for treating MASH patients with fibrosis stage 2–3,^7^,^8^ accurate risk stratification to identify potentially eligible patients has become clinically critical.

Although liver biopsy remains the gold standard for diagnosing MASH, its invasive nature, sampling variability, and cost have limited its use.^9^ Consequently, noninvasive tests (NITs) are increasingly used to identify patients with at-risk MASH, those who are most likely to experience disease progression and, therefore, most likely to benefit from therapeutic intervention.^10^ NITs such as magnetic resonance elastography (MRE)-assessed liver stiffness,^11^ vibration-controlled transient elastography (VCTE)-assessed liver stiffness,^12^ MRI-AST (MAST) score,^13^ and FIB-4 index^14^ have shown promise for risk stratification.^15^ However, definitions of “at-risk MASH” vary across studies. Some require a diagnosis of MASH with fibrosis stage ≥2,^11^,^16^ while others also require a NAFLD Activity Score (NAS) ≥4 with at least one point each in steatosis, lobular inflammation, and hepatocellular ballooning.^13^,^17^,^18^ These inconsistencies make it difficult to directly compare diagnostic performance across studies and complicate clinical risk classification. Importantly, current guidelines for resmetirom treatment eligibility rely on a diagnosis of MASH with fibrosis stages 2–3,^19^ without NAS criteria. As a result, aligning a standardized, clinically meaningful definition of at-risk MASH with existing treatment guidelines is essential. It would improve diagnostic consistency, enhance the utility of NITs, and ensure that appropriate patients are identified for therapy.

To support clinical practice, the American Association for the Study of Liver Diseases (AASLD) guidelines recommended liver stiffness thresholds of ≥3.1 and ≤4.4 kPa by MRE or ≥8 and ≤15 kPa by VCTE to determine resmetirom treatment eligibility.^19^ However, real-world findings from the MAESTRO-NASH trial showed that nearly half of biopsy-proven F2-F3 patients fell outside these ranges.^20^

This prospective study aimed to standardize the definition of at-risk MASH and propose optimized MRE-based liver stiffness cutoffs to improve noninvasive risk stratification and expand access to resmetirom and semaglutide therapy.

## METHODS

### Participants

This study contained 2 prospective cohort studies collected at Mayo Clinic (clinical trial, NCT02565446) and the University of California San Diego (UCSD). A total of 413 participants who had suspected or diagnosed MASLD, underwent MRI/MRE exams and liver biopsies, were included. As this is a secondary analysis, only participants with complete MRI/MRE and histological data were included; therefore, no imputation for missing data was performed. All activities related to human participants were conducted in accordance with both the Declarations of Helsinki and Istanbul. The study was approved by the Mayo Clinic Institutional Review Board and the University of California San Diego Institutional Review Board, and written informed consent was obtained from all participants.

### Clinical and laboratory data

For both Mayo Clinic and UCSD cohorts, all participants underwent physical examinations and laboratory assessments at study entry. The sex, age, and body mass index were recorded. All laboratory tests were conducted by a standard method, including AST, ALT, and platelet count. The FIB-4 index was calculated based on the published formula,^21^ FIB-4 = (age [years] × AST [U/L]) / (platelet count [10^9^/L] × ALT [U/L]^1/2^).

### Histological assessment

All participants underwent liver biopsies. At each site, a single experienced pathologist evaluated the biopsy specimens while blinded to clinical and imaging data. Histological assessment followed the MASH Clinical Research Network criteria^22^ to standardize the diagnosis of MASH and to grade steatosis, lobular inflammation, hepatocellular ballooning, and stage fibrosis. MASH was defined as the presence of hepatic steatosis (≥5% of hepatocytes) accompanied by hepatocellular ballooning and lobular inflammation on liver biopsy, irrespective of fibrosis stage. At-risk MASH was defined in 2 ways: definition 1, MASH with fibrosis stage ≥2; definition 2: in addition to meeting definition 1, the biopsy must also show steatosis, inflammation, and ballooning scores each ≥1, with NAS ≥4.

### MR image assessment

Conventional MRI/MRE exams were performed and analyzed at both sites as described^11^,^15^ on 1.5T or 3.0T systems (GE Healthcare, Waukesha, WI). All patients were scanned in the supine position after a fasting period of at least 4 hours. The image protocol contains MR Elastography (60 Hz gradient-echo 2D MRE, detailed MRE acquisition parameters are provided in Supplemental Material S1, https://links.lww.com/HC9/C453), which generated parametric stiffness maps called elastograms, and confounder-corrected chemical-shift-encoded imaging (IDEAL-IQ), which generated proton density fat fraction (PDFF) parametric maps. The liver stiffness (LS-MRE) and PDFF values were measured from the elastograms and PDFF maps, respectively, by image analysts who were blinded to all clinical and histological information at both sites. Regions of interest for LS-MRE were placed in the right hepatic lobe, avoiding major vessels, bile ducts, and areas of artifact, and were guided by confidence masks to ensure reliable measurements. Only regions with adequate wave propagation and signal quality were included, and slices with poor image quality were excluded from analysis. PDFF was calculated as the mean value of segmental regions of interest with weighting based on the regions of interest size.

### Statistical analysis

FIB-4 index and MAST score were calculated according to previously reported methods.^18^,^23^ Differences between the Mayo Clinic (derivation cohort) and UCSD (validation cohort) datasets were assessed using the Wilcoxon rank sum test for continuous variables and Fisher exact test for categorical variables. Logistic regression models based on LS-MRE, MAST score, and the combination of LS-MRE and FIB-4 were developed to predict at-risk MASH using definition 1, and separately using definition 2, in both the derivation and validation cohorts. Calibration performance of LS-MRE models was evaluated using calibration plots and Brier scores. In the combined (derivation plus validation) cohort, in order to test all potential models, additional imaging-based or nonimaging-based NITs, individually or in combination, were developed to predict at-risk MASH and cirrhosis. Model performance was compared to LS-MRE alone using the paired DeLong test.

To assess clinical decision-making for resmetirom and semaglutide therapy, we evaluated the diagnostic performance of guideline-recommended cutoffs of 3.1 and 4.4 kPa.^19^ To align with the guidelines using LS-MRE as a single criterion for treatment eligibility for MASH with moderate-advanced fibrosis, we derived optimized LS-MRE cutoffs that identify at-risk MASH (definition 1) while excluding cirrhosis. Our goal was to enable identification of all potential candidates,^24^ aligning with AASLD guidelines aimed at broadening the eligible population.^19^ To achieve this goal, we selected a lower cutoff with 90% sensitivity for at-risk MASH (values below this level would rule out at-risk MASH with high NPV) and a higher cutoff with 98% specificity for cirrhosis (values above this level would rule in cirrhosis with high PPV).^25^ In this way, we would exclude candidates who are very unlikely to have at-risk MASH as well as those who are very likely to have cirrhosis. The diagnostic accuracy for identifying pharmacotherapy eligibility of optimized LS-MRE cutoffs was compared with the AASLD guideline-recommended cutoffs.^19^

Decision curve analysis was performed to evaluate the clinical utility of the optimized and guideline-recommended LS-MRE cutoffs across threshold probabilities ranging from 0.05 to 0.50, representing clinically plausible decision thresholds for treatment consideration. Net reclassification improvement (NRI) was calculated to assess improvement in classification performance of the optimized cutoffs relative to guideline-recommended cutoffs. A significance level of *p*<0.05 was used in this study. All statistical analyses were performed using R (version 2024.12.0).

## RESULTS

### Participant characteristics

In this prospective study, 165 participants were enrolled from Mayo Clinic, as the derivation cohort, and 248 from UCSD, as the validation cohort. Table 1 shows the clinical, imaging, and histological characteristics of participants of both cohorts. Participants in the derivation cohort were significantly younger (median: 50 vs. 54 y, *p*=0.005) and had higher body mass index (median: 40 vs. 31 kg/m^2^, *p*<0.001). Both cohorts had a female-dominated sex distribution, with a higher proportion of females in the derivation cohort (68% vs. 57%, *p*=0.02). The racial distribution differed between cohorts, with more Caucasian participants in the derivation cohort and more Asian and Hispanic participants in the validation cohort, whereas Black and other racial groups were comparable. The prevalence of type 2 diabetes was comparable between the 2 cohorts (32% vs. 40%, *p*=0.12). Laboratory values (AST, ALT, platelet count) and FIB-4 score were significantly higher in the validation cohort. Histologically, participants in the validation cohort had more advanced steatosis and lobular inflammation (*p*<0.001 for both), while fibrosis stage and ballooning grade, and the prevalence of MASH, at-risk MASH using definition 1, at-risk MASH using definition 2, and eligibility for pharmacotherapy (MASH + F2-3) were comparable between cohorts. The median MRI-biopsy time interval in the overall cohort was 19 days (IQR: 6–50 d).

**TABLE 1:** Characteristics of participants

	Mayo Clinic (derivation)	UCSD (validation)	*p*
Demographics			
Total no. participants	165	248	
Sex (females)	113 (68)	142 (57)	0.02*
Age (y)	50 (41, 58)	54 (43, 64)	0.005*
BMI (kg/m^2^)	40 (33, 46)	31 (28, 35)	<0.001*
Race/ethnicity			<0.001*
Caucasian	148 (90)	106 (43)	
Black/African American	3 (2)	6 (2)	
Hispanic	7 (4)	75 (30)	
Asian	2 (1)	48 (19)	
Other	5 (3)	13 (5)	
Metabolic			
Type 2 diabetes (yes)	53 (32)	100 (40)	0.12
Blood			
AST (U/L)	29 (22, 40)	35 (26, 49)	<0.001*
ALT (U/L)	36 (23, 63)	48 (34, 72)	<0.001*
Platelets count (×10^9^/L)	250 (211, 295)	227 (183, 281)	0.003*
FIB-4 score	0.97 (0.67, 1.46)	1.22 (0.80, 1.89)	<0.001*
Histological findings			
Fibrosis stage			0.29
0	71 (43)	89 (36)	
1	39 (24)	79 (32)	
2	25 (15)	29 (12)	
3	16 (10)	25 (10)	
4	14 (8)	26 (10)	
Steatosis grade			<0.001*
0	34 (21)	16 (6)	
1	82 (50)	118 (48)	
2	35 (21)	83 (33)	
3	14 (8)	31 (13)	
Lobular inflammation grade			<0.001*
0	48 (29)	18 (7)	
1	108 (65)	143 (58)	
2	9 (5)	80 (32)	
3	0	7 (3)	
Ballooning grade			0.93
0	76 (46)	109 (44)	
1	72 (44)	113 (46)	
2	17 (10)	26 (10)	
MASH	93 (56)	155 (63)	0.22
At-risk MASH			
D1: MASH + F≥2	45 (27)	77 (31)	0.44
D2: MASH + F≥2 + S/I/B≥1 + NAS≥4	24 (15)	53 (21)	0.09
MASH + F2-3	36 (22)	52 (21)	0.90
Imaging findings			
Liver stiffness (kPa)	2.7 (2.2, 3.5)	2.5 (2.1, 3.4)	0.14
PDFF (%)	14 (7, 21)	12 (7, 19)	0.13

*Note:* Continuous variables are presented as median (IQR), and categorical variables as number (%).

* *p*<0.05.

Abbreviations: BMI, body mass index; AST, aspartate aminotransferase; ALT, alanine aminotransferase; MASH, metabolic dysfunction–associated steatohepatitis; ≥F2, fibrosis stage 2 or higher; S/I/B≥1, steatosis, inflammation, and ballooning grades all equal or higher than 1; NAS, nonalcoholic fatty liver disease activity score; PDFF, proton density fat fraction.

### Diagnostic performance of liver stiffness and related models for identifying at-risk MASH in the derivation cohort

**FIGURE 1:**
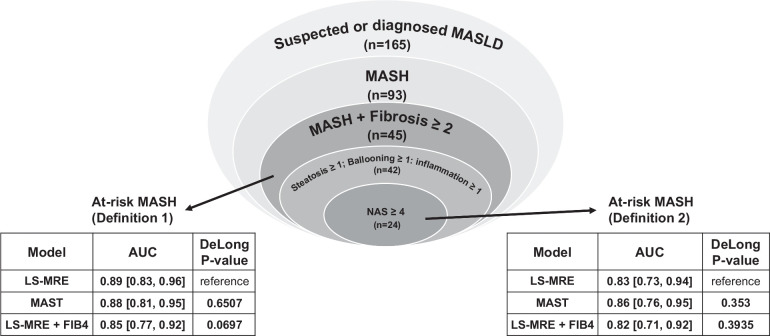
Diagnostic performance of models in the derivation cohort. Abbreviations: MASLD, metabolic dysfunction–associated liver disease; MASH, metabolic dysfunction–associated steatohepatitis; NAS, NAFLD activity score; LS-MRE, MRE-assessed liver stiffness; MAST, magnetic resonance imaging–aspartate aminotransferase score.

Figure 1 illustrates the diagnostic performance of different LS-MRE-based models for identifying at-risk MASH using each definition in the derivation cohort. Using definition 1 (MASH + F≥2), 45 of 165 (27%) participants had at-risk MASH based on the histology reference standard. Among the models, LS-MRE had the highest AUC point estimate (0.89 [0.83, 0.96]), followed by the MAST score (AUC =0.88 [0.81, 0.95]) and a regression model combining LS-MRE with FIB-4 (AUC =0.85 [0.77, 0.92]) (MEFIB equivalent). Using definition 2 (MASH + F≥2 + S/I/B≥1 + NAS≥4), 24 of 165 (15%) participants had at-risk MASH. The MAST had the highest AUC point estimate (AUC =0.86 [0.76, 0.95]), followed by LS-MRE (AUC =0.83 [0.73–0.94] and LS-MRE with FIB-4 (AUC =0.82 [0.71, 0.92]. However, no pairwise differences in AUC between models were significant using either definition.

### Diagnostic performance of liver stiffness and related models for identifying at-risk MASH in the validation cohort

**FIGURE 2:**
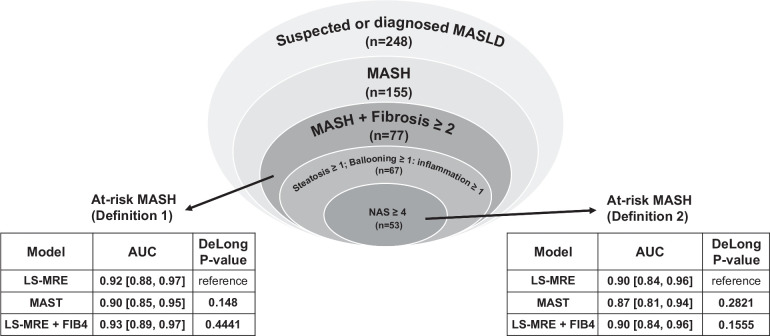
Diagnostic performance of models in the validation cohort. Abbreviations: MASLD, metabolic dysfunction–associated liver disease; MASH, metabolic dysfunction–associated steatohepatitis; NAS, NAFLD activity score; LS-MRE, MRE-assessed liver stiffness; MAST, magnetic resonance imaging–aspartate aminotransferase score.

As shown in Figure 2, in the validation cohort, 77/248 (31%) participants had at-risk MASH based on definition 1. The LS-MRE + FIB-4 model had the highest AUC point estimate (0.93 [0.89, 0.97]), followed by LS-MRE alone (0.92 [0.88, 0.97]) and the MAST score (0.90 [0.85, 0.95]). When applying definition 2, 53/248 (21%) participants had at-risk MASH. Both LS-MRE and the LS-MRE + FIB-4 model had an AUC of 0.90 [0.84, 0.96], while MAST had an AUC of 0.87 [0.81, 0.94]. Similar to the derivation cohort analysis, no pairwise differences in AUC between models were significant using either definition.

### Comparisons between imaging-based parameters and other NIT in identifying patients with at-risk MASH

In the combined cohort, 122/413 (30%) participants had at-risk MASH (definition 1, MASH + F≥2) (Table 2, results of definition 2 presented in Supplemental Material S2, https://links.lww.com/HC9/C453). LS-MRE demonstrated excellent diagnostic performance for identifying at-risk MASH (AUC =0.91 [0.87, 0.95]), significantly outperforming PDFF and all nonimaging NITs (*p*<0.01 for all). Combining LS-MRE with PDFF or other nonimaging parameters did not improve the diagnostic performance (*p*>0.05 for all).

**TABLE 2:** Diagnostic performance of imaging-based parameters and other noninvasive liver disease assessments (NITs) for at-risk MASH or cirrhosis

Models	At-risk MASH (MASH + F≥2)		Cirrhosis	
	AUC	*p*	AUC	*p*
Imaging-based NITs				
LS-MRE	0.91 [0.87, 0.95]	Ref	0.93 [0.88, 0.99]	Ref
PDFF	0.51 [0.45, 0.57]	<0.001*	0.73 [0.64, 0.82]	<0.001*
LS-MRE + PDFF	0.91 [0.87, 0.94]	0.85	0.94 [0.88, 0.99]	0.87
Nonimaging NITs				
AST	0.74 [0.69, 0.80]	<0.001*	0.62 [0.53, 0.72]	<0.001*
ALT	0.64 [0.58, 0.70]	<0.001*	0.52 [0.43, 0.62]	<0.001*
PLT	0.66 [0.60, 0.72]	<0.001*	0.76 [0.68, 0.85]	<0.001*
AST+PLT	0.77 [0.71, 0.82]	<0.001*	0.78 [0.69, 0.87]	<0.001*
FIB-4	0.78 [0.73, 0.84]	<0.001*	0.84 [0.77, 0.92]	0.002*
Combined (imaging + nonimaging) NIT Models				
MAST	0.89 [0.85, 0.93]	0.18	0.86 [0.79, 0.94]	<0.001*
LS-MRE + FIB4	0.90 [0.86, 0.94]	0.26	0.94 [0.88, 0.99]	0.85
LS-MRE + AST + PLT	0.91 [0.87, 0.94]	0.73	0.93 [0.87, 0.98]	0.64
LS-MRE + PDFF + PLT	0.91 [0.87, 0.94]	0.77	0.93 [0.88, 0.99]	0.87
LS-MRE + PDFF + AST	0.91 [0.87, 0.95]	0.80	0.94 [0.88, 0.99]	0.82
LS-MRE + PDFF + FIB4	0.90 [0.86, 0.94]	0.39	0.94 [0.89, 0.99]	0.68

* *p*<0.05.

Abbreviations: LS-MRE, MRE-assessed liver stiffness; PDFF, proton density fat fraction; PLT, platelet count.

### Comparisons between imaging-based parameters and other NITs in identifying patients with cirrhosis

In the combined cohort, 40/413 (10%) participants had cirrhosis. As shown in Table 2, LS-MRE had the highest AUC for identifying cirrhosis (0.93 [0.88, 0.99]), significantly outperforming PDFF and all nonimaging NITs (*p*<0.01 for all). Although combining LS-MRE with PDFF or FIB-4 increased the AUC point estimate (LS-MRE + PDFF: 0.94 [0.88, 0.99], LS-MRE + FIB4: 0.94 [0.88, 0.99]), the difference was not significant. Similarly, other models that integrated LS-MRE with nonimaging NITs did not improve performance significantly. MAST had significantly lower AUC (0.86 [0.79, 0.94]) than LS-MRE (*p*<0.01).

### Clinical application of liver stiffness in identifying patients suitable for resmetirom and semaglutide therapy

**FIGURE 3:**
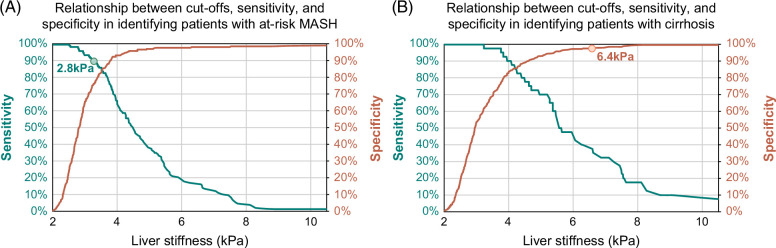
Relationship between MRE-assessed liver stiffness (LS-MRE) cutoffs, sensitivity, and specificity for identifying patients with at-risk MASH (MASH + F≥2) and cirrhosis. (A) An LS-MRE cutoff of 2.8 kPa achieves a sensitivity of 89% and specificity of 77% for identifying patients with at-risk MASH. (B) An LS-MRE cutoff of 6.4 kPa achieves a specificity of 98% and sensitivity of 33% for identifying patients with cirrhosis.

In the combined cohort, 88/413 (21%) participants were suitable for pharmacotherapy (MASH + F2-3) based on histology. Figure 3 illustrates the relationship between LS-MRE cutoff values, sensitivity, and specificity in identifying patients with at-risk MASH (definition 1, MASH + F≥2) and cirrhosis (model parameters are summarized in Supplemental Material S3, https://links.lww.com/HC9/C453). A lower LS-MRE cutoff of 2.8 kPa had 89% sensitivity, 77% specificity, and 94%NPV for identifying at-risk MASH. A higher cutoff of 6.4 kPa had 33% sensitivity, 98% specificity, and 65% PPV for detecting cirrhosis. Calibration analysis demonstrated good agreement between predicted and observed probabilities with low Brier scores (Supplemental Material S4, https://links.lww.com/HC9/C453).

**FIGURE 4:**
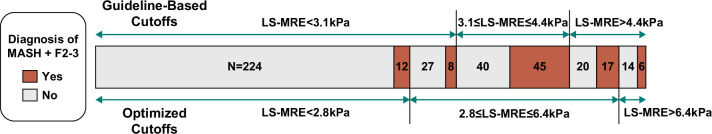
Patient distribution across MRE-assessed liver stiffness (LS-MRE) using AASLD guideline-recommended and optimized cutoffs for identifying patients eligible for resmetirom and semaglutide therapy (MASH + F2-3). Each box shows the number of patients histologically diagnosed with (orange) or without (gray) MASH + F2-3.

In Figure 4, the AASLD guideline-recommended cutoffs of 3.1 and 4.4 kPa identified 85/413 (21%) as being eligible for pharmacotherapy (MASH + F2-3),^19^ yielding 51% sensitivity, 88% specificity, 53% PPV, and 87% NPV (Table 3); 43/88 (49%) patients with MASH + F2-3 were falsely classified as being ineligible. In contrast, the optimized LS-MRE cutoffs of 2.8 and 6.4 kPa identified 157/413 (38%) as being eligible for pharmacotherapy. These cutoffs had 80% sensitivity, 73% specificity, 45% PPV, and 93% NPV; 18/88 (20%) patients with MASH + F2-3 were falsely classified as being ineligible. Notably, the optimized cutoffs identified an additional 25/88 (28%) treatment-eligible patients compared with the guideline-recommended cutoffs, consistent with their improved sensitivity. Histologic characteristics of FN and FP cases are summarized in Supplemental Material S5, https://links.lww.com/HC9/C453. Diagnostic performance of optimized cutoffs (2.8–6.4 kPa) in the derivation and validation cohorts is presented separately in Supplemental Material S6, https://links.lww.com/HC9/C453. Compared with guideline-recommended cutoffs, the optimized LS-MRE cutoffs demonstrated an NRI of 0.14, primarily driven by improved classification of patients eligible for pharmacotherapy (NRI_events =0.28), although this was partially offset by increased false-positive classification among nonevents (NRI_nonevents =−0.14).

**TABLE 3:** Diagnostic performance of guideline-recommended and optimized cutoffs for identifying patients eligible for resmetirom and semaglutide therapy in combined cohorts

Cutoffs	Sensitivity (%)	Specificity (%)	NPV (%)	PPV (%)	TP	FP	TN	FN
Guideline-recommended 3.1≤LS-MRE≤4.4 kPa	51	88	87	53	45	40	285	43
Optimized 2.8≤LS-MRE≤6.4 kPa	80	73	93	45	70	87	238	18

*Note:* n=413.

Abbreviations: FN, false negative; FP, false positive; NPV, negative predictive value; PPV, positive predictive value; TN, true negative; TP, true positive.

Head-to-head comparisons with the MEFIB index (MRE and FIB-4)^23^ and the M-PAST score^26^ are summarized in Supplemental Material S7, https://links.lww.com/HC9/C453. Overall, the optimized LS-MRE cutoffs demonstrated higher sensitivity with comparable negative predictive value, while MEFIB and M-PAST showed relatively higher specificity. Subgroup analyses stratified by body mass index, T2DM, sex, and ALT are presented in Supplemental Material S8, https://links.lww.com/HC9/C453.

**FIGURE 5:**
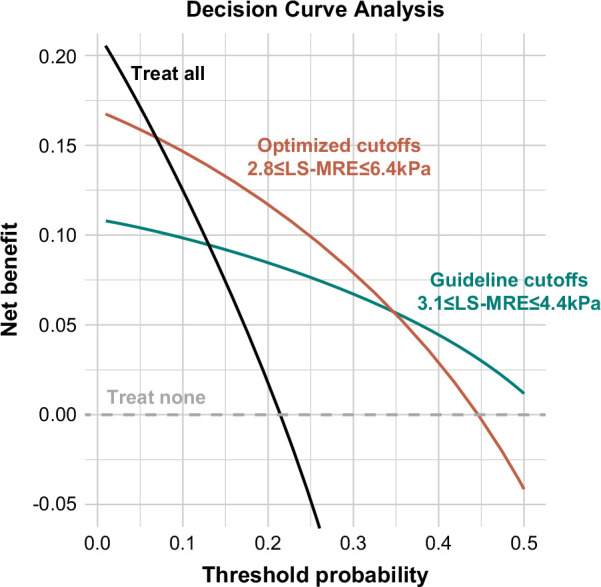
Decision curve analysis (DCA) comparing AASLD guideline-recommended and optimized LS-MRE cutoffs for identifying patients eligible for resmetirom and semaglutide therapy (MASH + F2-3).

Decision curve analysis demonstrated that both the AASLD guideline-recommended and the optimized LS-MRE cutoffs provided positive net benefit across a broad range of threshold probabilities, indicating clinical utility compared with default strategies of treating all or treating none (Figure 5).

## DISCUSSION

In this prospective, multicenter study, we found that using a simplified definition of at-risk MASH (MASH with fibrosis stage ≥2) enabled the identification of a broader group of patients requiring clinical attention, either for potential resmetirom and semaglutide therapy or for closer monitoring of disease progression. Among patients with suspected or diagnosed MASLD, a single parameter of LS-MRE demonstrated excellent diagnostic accuracy in identifying both at-risk MASH (AUC =0.91 [0.87, 0.95]) and cirrhosis (AUC =0.93 [0.88, 0.99]). Furthermore, applying a broader range of liver stiffness cutoffs (2.8 and 6.4 kPa), compared to the AASLD-recommended cutoffs (3.1 and 4.4 kPa), improved the ability to identify patients who may be eligible for pharmacotherapy.

Many studies have focused on the diagnosis of at-risk MASH; however, the definitions used vary across the literature, creating challenges for standardizing diagnostic criteria and assessing treatment eligibility. Some studies define at-risk MASH as the presence of MASH with fibrosis stages ≥2,^11^,^16^ while others use more strict criteria, requiring a NAS≥4 with at least one point each in steatosis, lobular inflammation, and hepatocellular ballooning.^13^,^17^,^18^ These varying definitions result in differences in patient inclusion and reported outcomes, complicating comparisons across studies. A unified, consensus definition is needed to enable parallel evaluation of diagnostic tools and therapeutic strategies. In our cohort (n=413), applying the stricter definition would have excluded 45 patients who otherwise met criteria based on fibrosis stage alone, including 28 individuals who fulfilled the AASLD guideline for resmetirom and semaglutide therapy (MASH + F2-3) (detailed information in Supplemental Material S9, https://links.lww.com/HC9/C453).^19^ This suggests that the stricter definition may exclude a substantial number of potential treatment candidates.

Furthermore, while inflammation and ballooning are key components of the stricter definition, their histological assessment is inherently subjective. One study reported that there was only slight to moderate (κ coefficient =0.13–0.45) intersample agreement for these features in paired biopsy specimens from the same individual.^27^ Therefore, relying on subjective features like inflammation and ballooning may lead to misclassification, whereas a simpler definition based on the presence of MASH with fibrosis stage ≥2 may offer a more reliable and clinically meaningful approach for identifying patients at risk and eligible for treatment.

The diagnostic accuracy of the models evaluated in this study is consistent with previous publications.^11^,^18^,^28^ We found that the MAST score demonstrated strong accuracy for identifying at-risk MASH in both the derivation (AUC =0.86 [0.76, 0.95]) and validation cohorts (AUC =0.87 [0.81, 0.94]), which closely aligns with the diagnostic performance reported in the literature (AUC =0.86 [0.776, 0.928]).^18^ The FIB-4 index showed moderate diagnostic performance in our analysis (AUC =0.78 [0.73, 0.84]), slightly exceeding a prior study (AUC =0.68) for identifying at-risk MASH.^28^ This difference may be due to the fact that the prior study included a larger sample size with a higher proportion of patients with advanced fibrosis, which can reduce the sensitivity of FIB-4 in distinguishing intermediate stages.^29^ Among all the models, LS-MRE alone demonstrated the highest and most consistent diagnostic accuracy in identifying at-risk MASH. We further evaluated combined models incorporating LS-MRE with laboratory and imaging parameters (including FIB-4, AST, platelet count, and PDFF); however, these models did not demonstrate meaningful improvement in diagnostic performance compared with LS-MRE alone. LS-MRE achieved an AUC of 0.91 [0.87, 0.95] in our cohort (n=413), which is comparable to results from a smaller cohort (n=104) that reported an AUC of 0.89 [0.82, 0.95].^11^ These findings highlight the robustness of LS-MRE as a reliable single-parameter tool for noninvasive risk stratification of MASLD. Nevertheless, liver stiffness may be influenced by confounding factors such as acute inflammation, hepatic congestion, and cholestasis. Emerging MRE-derived biomarkers, including tissue heterogeneity^30^ and loss modulus,^31^ may provide complementary information regarding inflammatory or vascular-related changes beyond fibrosis alone. Future studies integrating these advanced MRE biomarkers together with clinical history and laboratory findings may enable more individualized and biologically specific risk stratification.

With the approval of resmetirom and semaglutide, the most recent AASLD practice guidelines recommend using more accurate imaging-based NITs, such as liver stiffness measurement by VCTE or MRE, to determine treatment eligibility. LS-MRE between 3.1 and 4.4 kPa have been proposed as cutoffs for identifying eligible patients.^19^ However, in our cohort, applying these guideline-recommended cutoffs yielded a sensitivity of only 51% and specificity of 88%, identifying just 45 candidates for resmetirom therapy.

Recent expert recommendations published in *Clinical Gastroenterology and Hepatology* (CGH) have proposed a slightly wider treatment window of 3.3–5.0 kPa for identifying patients with at-risk MASH who may benefit from therapy (MASH + F2-3).^32^,^33^ When applied to our cohort, the CGH cutoffs showed diagnostic performance comparable to the AASLD cutoffs and identified a similar proportion of treatment-eligible patients (Supplemental Material S10, https://links.lww.com/HC9/C453). However, both guideline-recommended approaches missed nearly half of biopsy-confirmed at-risk MASH cases in this cohort.

To improve identification of treatment-eligible patients, we optimized the stiffness cutoffs to identify patients with at-risk MASH while excluding those with cirrhosis. The lower cutoff was chosen to achieve 89% sensitivity with 94% NPV, effectively ruling out patients with minimal likelihood of at-risk MASH. The higher cutoff was set with extremely high specificity (98%) to confidently exclude patients with a high likelihood of having cirrhosis, yielding a PPV of 65%.^25^ Since screening for cirrhosis should prioritize specificity over sensitivity to minimize false positives, this approach ensures that patients ruled out truly have cirrhosis. In other words, it helps provide treatment to all potential candidates,^24^ aligning with AASLD guidelines aimed at broadening the eligible cohort.^19^ Applying this optimized cutoff allowed identification of 25 additional biopsy-proven candidates, 28% of all candidates (25 of 88), which is a clinically significant improvement. This improved sensitivity comes with a modest reduction in PPV compared with the guideline-recommended thresholds, reflecting the trade-off inherent in prioritizing detection of treatment-eligible patients. Given the relatively low prevalence of at-risk MASH in this cohort (21%), moderate PPV values are expected for screening strategies designed to minimize missed cases. Although the optimized LS-MRE cutoffs increased false-positive classifications, many of these patients had compensated cirrhosis with ongoing steatohepatitis activity, a population that may potentially benefit from future expansion of pharmacotherapy indications.^34^ In addition, many false-positive cases with lower fibrosis stages may reflect biopsy sampling variability related to heterogeneous distribution of steatosis and fibrosis within the liver.^27^ Because LS-MRE reflects liver stiffness averaged over a substantially larger liver volume, some of these patients may have had more advanced fibrosis than indicated by the biopsy findings. Notably, many of these cases still demonstrated active steatohepatitis features, including steatosis, inflammation, and ballooning, suggesting biologically active disease that may warrant closer monitoring or therapeutic consideration.

This study has several limitations. First, although MRE postprocessing was centralized to ensure uniform image analysis, liver biopsy specimens were interpreted by local pathologists at each site using standard histological criteria without centralized or double reading. This may introduce interobserver variability, particularly in the assessment of steatohepatitis features such as hepatocellular ballooning and lobular inflammation. Second, the cohort had a relatively mild disease burden. While this may limit generalizability to more advanced populations, it reflects typical patients seen in clinical practice, where early-stage disease is more common.^35^,^36^ Third, we did not have VCTE data available, so we could not calculate or compare our results with other widely used models, such as the FibroScan-AST (FAST) score,^13^ which incorporates VCTE and is often used in noninvasive assessment of MASLD.

In conclusion, this study demonstrates that a simplified definition of at-risk MASH and the use of MRE-assessed liver stiffness can effectively identify patients who may benefit from resmetirom and semaglutide therapy. The excellent diagnostic performance of liver stiffness, along with the application of broader, optimized cutoffs, enhances the identification of eligible candidates for resmetirom and semaglutide therapy. These findings support the use of MRE as a noninvasive, reliable tool to guide clinical decision-making and optimize patient selection for emerging therapies.

## Supplementary Material

## References

[1] Rinella ME Lazarus JV Ratziu V Francque SM Sanyal AJ Kanwal F . A multisociety Delphi consensus statement on new fatty liver disease nomenclature. J Hepatol. 2023;79:1542–1556. 37364790 10.1016/j.jhep.2023.06.003

[2] Younossi ZM Golabi P Paik JM Henry A Van Dongen C Henry L . The global epidemiology of nonalcoholic fatty liver disease (NAFLD) and nonalcoholic steatohepatitis (NASH): a systematic review. Hepatology. 2023;77:1335–1347. 36626630 10.1097/HEP.0000000000000004 PMC10026948

[3] Huang DQ Wong VWS Rinella ME Boursier J Lazarus JV Yki-Järvinen H . Metabolic dysfunction-associated steatotic liver disease in adults. Nat Rev Dis Primers. 2025;11:14. 40050362 10.1038/s41572-025-00599-1

[4] Kanwal F Neuschwander-Tetri BA Loomba R Rinella ME . Metabolic dysfunction–associated steatotic liver disease: Update and impact of new nomenclature on the American Association for the Study of Liver Diseases practice guidance on nonalcoholic fatty liver disease. Hepatology. 2024;79:1212–1219. 38445559 10.1097/HEP.0000000000000670 PMC13435502

[5] Rinella ME Neuschwander-Tetri BA Siddiqui MS Abdelmalek MF Caldwell S Barb D . AASLD Practice Guidance on the clinical assessment and management of nonalcoholic fatty liver disease. Hepatology. 2023;77:1797–1835. 36727674 10.1097/HEP.0000000000000323 PMC10735173

[6] Estes C Razavi H Loomba R Younossi Z Sanyal AJ . Modeling the epidemic of nonalcoholic fatty liver disease demonstrates an exponential increase in burden of disease. Hepatology. 2018;67:123–133. 28802062 10.1002/hep.29466 PMC5767767

[7] Rezdiffra . Prescribing information. Madrigal Pharmaceuticals. Accessed June 17, 2024. https://www.madrigalpharma.com/wp-content/uploads/2024/06/NDA-217785_REZDIFFRA-PI_14Mar2024_final-revised-clean-SPLPPI.pdf

[8] Sanyal AJ Newsome PN Kliers I Østergaard LH Long MT Kjær MS . Phase 3 trial of semaglutide in metabolic dysfunction-associated steatohepatitis. N Engl J Med. 2025;392:2089–2099. 40305708 10.1056/NEJMoa2413258

[9] Sumida Y Nakajima A Itoh Y . Limitations of liver biopsy and non-invasive diagnostic tests for the diagnosis of nonalcoholic fatty liver disease/nonalcoholic steatohepatitis. World J Gastroenterol. 2014;20:475–485. 24574716 10.3748/wjg.v20.i2.475 PMC3923022

[10] Tincopa MA Loomba R . Non-invasive diagnosis and monitoring of non-alcoholic fatty liver disease and non-alcoholic steatohepatitis. Lancet Gastroenterol Hepatol. 2023;8:660–670. 37060912 10.1016/S2468-1253(23)00066-3

[11] Li J Lu X Zhu Z Kalutkiewicz KJ Mounajjed T Therneau TM . Head-to-head comparison of magnetic resonance elastography-based liver stiffness, fat fraction, and T1 relaxation time in identifying at-risk NASH. Hepatology. 2023;78:1200–1208. 37080558 10.1097/HEP.0000000000000417 PMC10521779

[12] Lee YK Lee DH Joo SK Jang H So YH Jang S . Combi-elastography versus transient elastography for assessing the histological severity of metabolic dysfunction-associated steatotic liver disease. Gut Liver. 2024;18:1048–1059. 39469729 10.5009/gnl240198 PMC11565001

[13] Imajo K Saigusa Y Kobayashi T Nagai K Nishida S Kawamura N . Head-to-head comparison among FAST, MAST, and multiparametric MRI-based new score in diagnosing at-risk MASH. Eur Radiol. 2025;35:3599–3609. 39638942 10.1007/s00330-024-11215-3

[14] Vali Y Lee J Boursier J Petta S Wonders K Tiniakos D . Biomarkers for staging fibrosis and non-alcoholic steatohepatitis in non-alcoholic fatty liver disease (the LITMUS project): a comparative diagnostic accuracy study. Lancet Gastroenterol Hepatol. 2023;8:714–725. 36958367 10.1016/S2468-1253(23)00017-1

[15] Kim BK Tamaki N Imajo K Yoneda M Sutter N Jung J . Head-to-head comparison between MEFIB, MAST, and FAST for detecting stage 2 fibrosis or higher among patients with NAFLD. J Hepatol. 2022;77:1482–1490. 35973577 10.1016/j.jhep.2022.07.020

[16] Adams LA Thiele M Tsochatzis EA . Detecting at-risk steatotic liver disease and liver fibrosis in the community. Hepatology. 2025. doi:10.1097/hep.0000000000001400 40384127

[17] Andersson A Kelly M Imajo K Nakajima A Fallowfield JA Hirschfield G . Clinical utility of magnetic resonance imaging biomarkers for identifying nonalcoholic steatohepatitis patients at high risk of progression: a multicenter pooled data and meta-analysis. Clin Gastroenterol Hepatol. 2022;20:2451–2461.e3. 34626833 10.1016/j.cgh.2021.09.041

[18] Noureddin M Truong E Gornbein JA Saouaf R Guindi M Todo T . MRI-based (MAST) score accurately identifies patients with NASH and significant fibrosis. J Hepatol. 2022;76:781–787. 34798176 10.1016/j.jhep.2021.11.012

[19] Chen VL Morgan TR Rotman Y Patton HM Cusi K Kanwal F . Resmetirom therapy for metabolic dysfunction-associated steatotic liver disease: October 2024 updates to AASLD Practice Guidance. Hepatology. 2025;81:312–320. 39422487 10.1097/HEP.0000000000001112

[20] Harrison SA Bedossa P Guy CD Schattenberg JM Loomba R Taub R . A phase 3, randomized, controlled trial of resmetirom in NASH with liver fibrosis. N Engl J Med. 2024;390:497–509. 38324483 10.1056/NEJMoa2309000

[21] Sterling RK Lissen E Clumeck N Sola R Correa MC Montaner J . Development of a simple noninvasive index to predict significant fibrosis in patients with HIV/HCV coinfection. Hepatology. 2006;43:1317–1325. 16729309 10.1002/hep.21178

[22] Kleiner DE Brunt EM Van Natta M Behling C Contos MJ Cummings OW . Design and validation of a histological scoring system for nonalcoholic fatty liver disease. Hepatology. 2005;41:1313–1321. 15915461 10.1002/hep.20701

[23] Jung J Loomba RR Imajo K Madamba E Gandhi S Bettencourt R . MRE combined with FIB-4 (MEFIB) index in detection of candidates for pharmacological treatment of NASH-related fibrosis. Gut. 2021;70:1946–1953. 33214165 10.1136/gutjnl-2020-322976 PMC8131405

[24] Grimes DA Schulz KF . Uses and abuses of screening tests. The Lancet. 2002;359:881–884. 10.1016/S0140-6736(02)07948-5 11897304

[25] Ochi H Hirooka M Koizumi Y Miyake T Tokumoto Y Soga Y . Real-time tissue elastography for evaluation of hepatic fibrosis and portal hypertension in nonalcoholic fatty liver diseases. Hepatology. 2012;56:1271–1278. 22488593 10.1002/hep.25756

[26] Imajo K Saigusa Y Kobayashi T Nagai K Nishida S Kawamura N . M-PAST score is better than MAST score for the diagnosis of active fibrotic nonalcoholic steatohepatitis. Hepatol Res. 2023;53:844–856. 37237426 10.1111/hepr.13927 PMC10792544

[27] Ratziu V Charlotte F Heurtier A Gombert S Giral P Bruckert E . Sampling variability of liver biopsy in nonalcoholic fatty liver disease. Gastroenterology. 2005;128:1898–1906. 15940625 10.1053/j.gastro.2005.03.084

[28] Mózes FE Lee JA Vali Y Selvaraj EA Jayaswal ANA Boursier J . Diagnostic accuracy of non-invasive tests to screen for at-risk MASH-An individual participant data meta-analysis. Liver Int. 2024;44:1872–1885. 38573034 10.1111/liv.15914

[29] Loomba R Adams LA . Advances in non-invasive assessment of hepatic fibrosis. Gut. 2020;69:1343–1352. 32066623 10.1136/gutjnl-2018-317593 PMC7945956

[30] Wu H Zhu Z Li J Qiu C Xu P Glaser KJ . Three-dimensional vector MR elastography for evaluating tissue mechanical heterogeneity to assess liver disease progression. Radiology. 2025;315:e242349. 40167439 10.1148/radiol.242349 PMC12784277

[31] Li J Allen AM Shah VH Manduca A Ehman RL Yin M . Longitudinal changes in MR elastography-based biomarkers in obese patients treated with bariatric surgery. Clin Gastroenterol Hepatol. 2023;21:220–222.e3. 34757198 10.1016/j.cgh.2021.10.033 PMC9054942

[32] Noureddin M Charlton MR Harrison SA Bansal MB Alkhouri N Loomba R . Expert panel recommendations: practical clinical applications for initiating and monitoring resmetirom in patients with MASH/NASH and moderate to noncirrhotic advanced fibrosis. Clin Gastroenterol Hepatol. 2024;22:2367–2377. 39038768 10.1016/j.cgh.2024.07.003

[33] Kim HY Rinella ME . Emerging therapies and real-world application of metabolic dysfunction-associated steatotic liver disease treatment. Clin Mol Hepatol. 2025;31:753–770. 40170239 10.3350/cmh.2025.0083 PMC12260617

[34] Magrigal Pharmaceuticals . Madrigal Announces New Two-Year Data from the Compensated MASH Cirrhosis Arm of the MAESTRO-NAFLD-1 Trial Demonstrating Potential Benefit of Rezdiffra™ (resmetirom) in Patients with Compensated MASH Cirrhosis. Accessed February 26, 2025. https://ir.madrigalpharma.com/news-releases/news-release-details/madrigal-announces-new-two-year-data-compensated-mash-cirrhosis

[35] Simeone J Bae J Hoogwerf B Li Q Haupt A Ali A . Clinical course of nonalcoholic fatty liver disease: an assessment of severity, progression, and outcomes. Clin Epidemiol. 2017;9:679–688. 29276410 10.2147/CLEP.S144368 PMC5733910

[36] Perumpail BJ Khan MA Yoo ER Cholankeril G Kim D Ahmed A . Clinical epidemiology and disease burden of nonalcoholic fatty liver disease. World J Gastroenterol. 2017;23:8263–8276. 29307986 10.3748/wjg.v23.i47.8263 PMC5743497

