# Supplementary material for: Simplified definition and imaging-based stratification of at-risk MASH using magnetic resonance elastography
Source: Hepatol Commun. 2026 Aug 11;10(9):e1024. doi: 10.1097/HC9.0000000000001024 (PMC13466114; doi:10.1097/HC9.0000000000001024)
Supplement: Supplementary file 1 [file hc9-10-e1024-s001.docx]

**Supplementary Material**

**S1. MRE acquisition parameters**

| Site | Mayo Clinic | UCSD |
| --- | --- | --- |
| Field strength | 1.5T | 3.0T |
| Sequence | Flow-compensated gradient-echo (GRE) MRE | Flow-compensated gradient-echo (GRE) MRE |
| Mechanical Frequencies | 60Hz | 60Hz |
| TR (ms) | 50 | 50 |
| TE (ms) | 18 | 20 |
| # of breath holds × breath holding time | 4 × 16s | 4 × 16s |
| Readout bandwidth (kHz) | 31.25 | 62.5 |
| Motion encoding directions | Z | Z |
| Phase Offsets | 4 | 4 |
| Number of Slices | 4 | 4 |
| Acquisition matrix | 256 × 64 × 4 | 256 × 64 × 4 |
| Recon matrix | 256 × 256 × 4 | 256 × 256 × 4 |
| Driver power (%) | 50 | 50 |
| Slice Thickness (mm) | 10 | 10 |
| Gap between slices | 0 | 0 |
| FOV (cm) | 32-48 | 32-48 |

*TR, repetition time; TE, echo time; FOV, field of view.*

**S2. Diagnostic performance of imaging-based parameters and other non-invasive liver disease assessments (NITs) for at-risk MASH (Definition 2)**

| Models | | At-risk MASH  (MASH + F≥2 + S/I/B≥1 + NAS≥4) | |
| --- | --- | --- | --- |
|  |  | AUC | p |
| Imaging-based NITs | **LS-MRE** | **0.87**  **[0.82, 0.92]** | **ref** |
|  | PDFF | 0.54  [0.46, 0.61] | <0.001* |
|  | LS-MRE+PDFF | 0.87  [0.81, 0.92] | 0.66 |
| Non-imaging NITs | AST | 0.74  [0.67, 0.81] | <0.001* |
|  | ALT | 0.65  [0.58, 0.72] | <0.001* |
|  | PLT | 0.63  [0.55, 0.70] | <0.001* |
|  | AST+PLT | 0.75  [0.68, 0.81] | <0.001* |
|  | FIB-4 | 0.75  [0.68, 0.82] | <0.001* |
| Combined (imaging + non-imaging)  NIT Models | MAST | 0.86  [0.81, 0.92] | 0.71 |
|  | LS-MRE + FIB4 | 0.87  [0.81, 0.92] | 0.65 |
|  | LS-MRE + AST + PLT | 0.86  [0.81, 0.92] | 0.56 |
|  | LS-MRE + PDFF + PLT | 0.86  [0.81, 0.92] | 0.45 |
|  | LS-MRE + PDFF + AST | 0.87  [0.81, 0.92] | 0.79 |
|  | LS-MRE + PDFF + FIB4 | 0.86  [0.81, 0.92] | 0.55 |

*LS-MRE, MRE-assessed liver stiffness; PDFF, proton density fat fraction; AST, aspartate aminotransferase; ALT, alanine aminotransferase;* PLT, platelet count.

**S3. Prediction models for identifying patients with at-risk MASH and cirrhosis**

|  | **At-risk MASH**  **(MASH + F≥2)** | **Cirrhosis** |
| --- | --- | --- |
| LS-MRE | $\frac{1}{1+e^{-(-4.8012+1.2462\times LS)}}$ | $\frac{1}{1+e^{-(-5.4740+0.85680\times LS)}}$ |
| MAST | $\frac{1}{1+e^{-(-1.9194+8.5721\times MAST)}}$ | $\frac{1}{1+e^{-(-3.1548+4.6875\times MAST)}}$ |
| LS-MRE + FIB4 | $\frac{1}{1+e^{-(-5.2604+0.93459\times LS+1.0121\times FIB4)}}$ | $\frac{1}{1+e^{-(-5.6806+0.69339\times LS+0.45236\times FIB4)}}$ |

**S4. Calibration plots for LS-MRE models in identifying patients with at-risk MASH (MASH + F≥2) and cirrhosis.** Calibration plots demonstrate agreement between predicted and observed probabilities. Brier scores were 0.119 for at-risk MASH and 0.060 for cirrhosis, indicating good overall calibration performance.

**S5. Histologic characteristics of false-negative (FN) and false-positive (FP) cases for Guideline-recommended (3.1≤LS-MRE≤4.4kPa) and Optimized (2.8≤LS-MRE≤6.4kPa) cutoffs**

A. **False-negative (FN) cases**

| **FN cases using Optimized cutoffs (2.8≤LS-MRE≤6.4kPa), n=18** | | | | | | | |
| --- | --- | --- | --- | --- | --- | --- | --- |
| Fibrosis stage |  | Steatosis grade |  | Inflammation grade |  | Ballooning grade |  |
| 0 |  | 0 |  | 0 |  | 0 | 3 |
| 1 |  | 1 | 6 | 1 | 13 | 1 | 10 |
| 2 | 12 | 2 | 6 | 2 | 5 | 2 | 5 |
| 3 | 6 | 3 | 6 | 3 |  |  |  |
| 4 |  |  |  |  |  |  |  |

Among the 18 FN cases identified using the optimized cutoffs, 6 cases had LS-MRE values above the upper threshold (>6.4 kPa). Of these, five had fibrosis stage 3 and one had fibrosis stage 2; all demonstrated steatosis, inflammation, and ballooning on histology. These findings may reflect heterogeneous distribution of histology and biopsy sampling variability in MASLD (1). Given that LS-MRE represents stiffness averaged over a substantially larger liver volume, the markedly elevated LS-MRE values in these cases may indicate more advanced fibrosis than suggested by the biopsy findings. The remaining 12 FN cases had LS-MRE values below the lower threshold (<2.8 kPa), consistent with the selection of the lower cutoff based on achieving 90% sensitivity for identifying at-risk MASH (MASH + F≥2).

| **FN cases reclassified by Optimized vs. Guideline cutoffs (2.8≤LS-MRE<3.1kPa), n=8** | | | | | | | |
| --- | --- | --- | --- | --- | --- | --- | --- |
| Fibrosis stage |  | Steatosis grade |  | Inflammation grade |  | Ballooning grade |  |
| 0 |  | 0 |  | 0 |  | 0 |  |
| 1 |  | 1 | 2 | 1 | 4 | 1 | 6 |
| 2 | 5 | 2 | 3 | 2 | 4 | 2 | 2 |
| 3 | 3 | 3 | 3 | 3 |  |  |  |
| 4 |  |  |  |  |  |  |  |
| **FN cases reclassified by Optimized vs. Guideline cutoffs (4.4<LS-MRE≤6.4kPa), n=17** | | | | | | | |
| Fibrosis stage |  | Steatosis grade |  | Inflammation grade |  | Ballooning grade |  |
| 0 |  | 0 |  | 0 |  | 0 |  |
| 1 |  | 1 | 13 | 1 | 8 | 1 | 9 |
| 2 | 6 | 2 | 2 | 2 | 8 | 2 | 8 |
| 3 | 11 | 3 | 2 | 3 | 1 |  |  |
| 4 |  |  |  |  |  |  |  |

Overall, the optimized cutoffs substantially reduced the number of FN cases and correctly identified an additional 25 treatment-eligible patients compared with the guideline-recommended cutoffs. These additionally identified patients all demonstrated steatosis, inflammation, and ballooning, with NAS ranging from 3–7, suggesting biologically active disease.

B. **False positive (FP) cases**

| **FP cases using Guideline-recommended cutoffs (3.1≤LS-MRE≤4.4kPa), n=40** | | | | | | | |
| --- | --- | --- | --- | --- | --- | --- | --- |
| Fibrosis stage |  | Steatosis grade |  | Inflammation grade |  | Ballooning grade |  |
| 0 | 7 | 0 | 4 | 0 | 7 | 0 | 13 |
| 1 | 21 | 1 | 24 | 1 | 26 | 1 | 24 |
| 2 |  | 2 | 10 | 2 | 6 | 2 | 3 |
| 3 | 2 | 3 | 2 | 3 | 1 |  |  |
| 4 | 10 |  |  |  |  |  |  |

Among FP cases identified using the guideline-recommended cutoffs, only one patient had completely negative biopsy findings (grade 0 fibrosis, steatosis, inflammation, and ballooning). Most FP cases (39/40) demonstrated at least some inflammatory activity, and 65% (26/40) had NAS≥3, suggesting ongoing disease activity and potential risk of progression. In addition, 28/40 cases had fibrosis stage 0–1, which may partially reflect biopsy sampling variability related to heterogeneous distribution of inflammation and fibrosis within the liver (1). Given that LS-MRE reflects liver stiffness averaged over a substantially larger liver volume, some of these cases may have had more advanced fibrosis than suggested by the biopsy findings.

| **FP cases reclassified by Optimized vs. Guideline cutoffs (2.8≤LS-MRE<3.1kPa), n=27** | | | | | | | |
| --- | --- | --- | --- | --- | --- | --- | --- |
| Fibrosis stage |  | Steatosis grade |  | Inflammation grade |  | Ballooning grade |  |
| 0 | 12 | 0 | 8 | 0 | 6 | 0 | 16 |
| 1 | 13 | 1 | 8 | 1 | 16 | 1 | 10 |
| 2 | 2 | 2 | 8 | 2 | 5 | 2 | 1 |
| 3 |  | 3 | 3 | 3 |  |  |  |
| 4 |  |  |  |  |  |  |  |
| **FP cases reclassified by Optimized vs. Guideline cutoffs (4.4<LS-MRE≤6.4kPa), n=20** | | | | | | | |
| Fibrosis stage |  | Steatosis grade |  | Inflammation grade |  | Ballooning grade |  |
| 0 |  | 0 | 1 | 0 |  | 0 |  |
| 1 | 3 | 1 | 12 | 1 | 14 | 1 | 13 |
| 2 |  | 2 | 7 | 2 | 5 | 2 | 7 |
| 3 | 1 | 3 |  | 3 | 1 |  |  |
| 4 | 16 |  |  |  |  |  |  |

Among FP cases identified at the lower LS-MRE range (2.8–3.1 kPa), only 4 patients had negative biopsy findings, while 18/27 had NAS≥3, indicating active disease. At the higher LS-MRE range (4.4–6.4 kPa), 16/20 had compensated cirrhosis and still demonstrated active steatosis, inflammation, and ballooning. Although these patients were classified as FP based on current treatment eligibility criteria, emerging evidence suggests that patients with compensated cirrhosis may also benefit from pharmacotherapy (2).

**S6. Diagnostic accuracy of the optimized LS-MRE cutoffs (2.8 and 6.4kPa) in identifying patients with MASH + F2-3 in derivation and validation cohorts**

|  | Number | Sensitivity | Specificity | NPV | PPV | TP | FP | TN | FN |
| --- | --- | --- | --- | --- | --- | --- | --- | --- | --- |
| Combined cohort | 413 | 80% | 73% | 93% | 45% | 70 | 87 | 238 | 18 |
| Derivation cohort | 165 | 78% | 70% | 92% | 42% | 28 | 39 | 90 | 8 |
| Validation cohort | 248 | 81% | 76% | 94% | 47% | 42 | 48 | 148 | 10 |

**S7. Diagnostic performance of the optimized LS-MRE cutoffs, MEFIB index, and M-PAST score for identifying patients with MASH + F2-3.**

| **MASH + F2-3** | **Sens.** | **Spec.** | **NPV** | **PPV** | **TP** | **FP** | **TN** | **FN** |
| --- | --- | --- | --- | --- | --- | --- | --- | --- |
| **Optimized LS-MRE**  **(**2.8≤LS-MRE≤6.4kPa**)** | 80% | 73% | 93% | 45% | 70 | 87 | 238 | 18 |
| **MEFIB index**  **(**LS-MRE<3.3kPa and FIB4<1.6/  Indeterminate/  LS-MRE≥3.3kPa and FIB4≥1.6**)** | 34% | 88% | 83% | 44% | 30 | 38 | 287 | 58 |
| **M-PAST score**  **(**LS-MRE≤3.0kPa or PAST≤0.165/  Indeterminate/  LS-MRE≥3.5kPa and PAST≥0.40**)** | 40% | 95% | 85% | 70% | 35 | 15 | 310 | 53 |

**S8. Diagnostic performance of the optimized LS-MRE cutoffs (2.8 and 6.4kPa) for identifying patients with MASH + F2-3 across subgroups.**

The diagnostic performance of the optimized LS-MRE cutoffs was consistent across clinically relevant subgroups defined by BMI, T2DM status, sex, and ALT levels. Notably, high NPV was maintained across all subgroups (89–98%), supporting the robustness of the model for ruling out at-risk MASH. Sensitivity was higher in patients with T2DM (85%) and in those with ALT <40 U/L (88%), while specificity was modestly reduced in these groups. PPV remained moderate across subgroups, reflecting the expected trade-off of a screening-oriented strategy. Overall, these findings demonstrate stable and clinically meaningful performance of LS-MRE–based stratification across diverse patient populations.

| **MASH + F2-3** | | **No.** | **Sens.** | **Spec.** | **NPV** | **PPV** | **TP** | **FP** | **TN** | **FN** |
| --- | --- | --- | --- | --- | --- | --- | --- | --- | --- | --- |
| **BMI** | **BMI<30kg/m^2^** | 107 | 78% | 79% | 93% | 50% | 18 | 18 | 66 | 5 |
|  | **BMI≥30kg/m^2^** | 306 | 80% | 71% | 93% | 43% | 52 | 69 | 172 | 13 |
| **T2DM** | **Not DM** | 260 | 73% | 81% | 94% | 41% | 29 | 42 | 178 | 11 |
|  | **DM** | 153 | 85% | 57% | 90% | 48% | 41 | 45 | 60 | 7 |
| **Sex** | **Female** | 255 | 81% | 74% | 93% | 49% | 48 | 50 | 146 | 11 |
|  | **Male** | 158 | 76% | 71% | 93% | 37% | 22 | 37 | 92 | 7 |
| **ALT** | **ALT<40U/L** | 182 | 88% | 75% | 98% | 35% | 21 | 39 | 119 | 3 |
|  | **ALT≥40U/L** | 231 | 77% | 71% | 89% | 51% | 49 | 48 | 119 | 15 |
| **Overall** | | **413** | **80%** | **73%** | **93%** | **45%** | **70** | **87** | **238** | **18** |

**S9. Comparison of at-risk MASH definitions across MRE-assessed liver stiffness thresholds: AASLD guideline-recommended vs. Optimized LS-MRE cutoffs**

Distribution of biopsy-proven at-risk MASH under two definitions across MRE-assessed liver stiffness (LS) thresholds:

1. Definition 1 (Top panel): MASH with fibrosis stage ≥2
2. Definition 2 (Bottom panel): MASH with fibrosis stage ≥2 plus NAS ≥4 and at least one point each in steatosis (S), inflammation (I), and ballooning (B)

LS ranges are segmented by two sets of thresholds:

1. Optimized cutoffs: 2.8 and 6.4 kPa
2. AASLD guideline-recommended cutoffs: 3.1 and 4.4 kPa

Each colored bar represents the number of patients falling within each LS range:

1. Orange: met the at-risk MASH definition
2. Gray: did not meet the definition
3. Blue: patients who met Definition 1 but not Definition 2

This figure illustrates how stricter definition of at-risk MASH (Definition 2) excluded a substantial portion of patients who meet the simplified definition (Definition 1), particularly within the clinically relevant LS ranges. Use of optimized LS thresholds may help identify more treatment-eligible patients without over-including cirrhosis cases.

Detailed histological diagnosis of individual patients is listed in following table.

|  | **ID** | **MASH (Yes 1/No 0)** | **Fibrosis stages** | **Steatosis grades** | **Lobular inflammation grades** | **Ballooning grade** | **NAS** |
| --- | --- | --- | --- | --- | --- | --- | --- |
| A | 1 | 1 | 2 | 1 | 1 | 1 | 3 |
|  | 2 | 1 | 2 | 3 | 1 | 0 | 4 |
|  | 3 | 1 | 2 | 1 | 1 | 1 | 3 |
|  | 4 | 1 | 2 | 3 | 1 | 0 | 4 |
|  | 5 | 1 | 2 | 1 | 1 | 1 | 3 |
|  | 6 | 1 | 4 | 1 | 1 | 1 | 3 |
|  | 7 | 1 | 2 | 2 | 2 | 0 | 4 |
| B | 8 | 1 | 2 | 1 | 1 | 0 | 3 |
| C | 9 | 1 | 2 | 1 | 1 | 1 | 3 |
|  | 10 | 1 | 4 | 1 | 1 | 1 | 3 |
|  | 11 | 1 | 2 | 1 | 1 | 1 | 3 |
|  | 12 | 1 | 2 | 1 | 1 | 1 | 3 |
|  | 13 | 1 | 3 | 1 | 1 | 1 | 3 |
|  | 14 | 1 | 4 | 1 | 0 | 1 | 2 |
|  | 15 | 1 | 2 | 3 | 1 | 1 | 5 |
|  | 16 | 1 | 3 | 1 | 0 | 0 | 1 |
|  | 17 | 1 | 4 | 1 | 1 | 1 | 3 |
|  | 18 | 1 | 2 | 1 | 1 | 1 | 3 |
|  | 19 | 1 | 3 | 1 | 1 | 1 | 3 |
|  | 20 | 1 | 2 | 1 | 1 | 1 | 3 |
|  | 21 | 1 | 4 | 1 | 0 | 1 | 2 |
|  | 22 | 1 | 4 | 1 | 1 | 1 | 3 |
|  | 23 | 1 | 2 | 1 | 1 | 1 | 3 |
|  | 24 | 1 | 2 | 1 | 1 | 0 | 2 |
|  | 25 | 1 | 2 | 1 | 1 | 1 | 3 |
|  | 26 | 1 | 4 | 1 | 1 | 1 | 3 |
|  | 27 | 1 | 2 | 1 | 1 | 1 | 3 |
|  | 28 | 1 | 3 | 1 | 1 | 1 | 3 |
|  | 29 | 1 | 3 | 1 | 2 | 0 | 3 |
|  | 30 | 1 | 2 | 1 | 1 | 1 | 3 |
| D | 31 | 1 | 2 | 1 | 1 | 1 | 3 |
|  | 32 | 1 | 4 | 1 | 1 | 1 | 3 |
|  | 33 | 1 | 4 | 1 | 1 | 1 | 3 |
|  | 34 | 1 | 4 | 0 | 1 | 1 | 2 |
|  | 35 | 1 | 2 | 1 | 1 | 1 | 3 |
|  | 36 | 1 | 4 | 1 | 1 | 1 | 3 |
|  | 37 | 1 | 2 | 1 | 1 | 1 | 3 |
|  | 38 | 1 | 4 | 1 | 1 | 1 | 3 |
|  | 39 | 1 | 3 | 1 | 1 | 1 | 3 |
| E | 40 | 1 | 4 | 1 | 1 | 1 | 3 |
|  | 41 | 1 | 3 | 1 | 1 | 1 | 3 |
|  | 42 | 1 | 4 | 0 | 0 | 0 | 0 |
|  | 43 | 1 | 4 | 0 | 2 | 2 | 4 |
|  | 44 | 1 | 4 | 1 | 1 | 1 | 3 |
|  | 45 | 1 | 4 | 0 | 1 | 2 | 3 |

From the table, we found that 34 patients were classified as non-at-risk MASH under Definition 2 because they had a NAS of 3. An additional 5 patients had NAS of 4, but one of the individual component scores (steatosis, inflammation or ballooning) was zero, excluding them from meeting Definition 2 criteria. Finally, 6 patients had NAS <3, also categorized as non-at-risk MASH under this definition.

Among these 45 patients, 28 would be eligible for resmetirom and semaglutide therapy under clinical practice guideline (MASH + F2-3). Their liver stiffness and NAS distribution were as follows:

A. LS<2.8kPa: 3 patients with NAS =3; 3 patients with NAS =4 but ballooning =0
B. 2.8≤LS<3.1kPa: 1 patient with NAS =3
C. 3.1≤LS≤4.4kPa: 13 patients with NAS =3, 1 patient with NAS =4 but inflammation =0, 2 patients with NAS =2
D. 4.4<LS≤6.4kPa: 4 patients with NAS =3
E. LS>6.4kPa: 1 patient with NAS =3

**S10. Patient distribution across MRE-assessed liver stiffness (LS-MRE) using AASLD, CGH guideline-recommended and optimized cutoffs for identifying patients eligible for resmetirom and semaglutide therapy (MASH + F2-3).** Each box shows the number of patients diagnosed with (orange) or without (grey) MASH + F2-3.

Using the CGH cutoffs (3.3–5.0 kPa), 83 of 413 patients (20%) were identified as treatment-eligible for MASH + F2-3, comparable to 85 of 413 patients (21%) identified using the AASLD cutoffs of 3.1–4.4 kPa. Diagnostic performance metrics were also similar between the two guideline-recommended approaches, including sensitivity, specificity, NPV, and PPV. However, both guideline-recommended intervals missed nearly half of the patients with biopsy-confirmed at-risk MASH. Specifically, 42 of 88 patients (48%) with MASH + F2-3 were classified as treatment-ineligible using the CGH cutoffs and 43 of 88 patients (49%) using the AASLD cutoffs. In contrast, the optimized LS-MRE interval identified a substantially larger proportion of patients (157 of 413, 38%) and reduced the false-negative rate, with only 18 of 88 patients (20%) with MASH + F2-3 classified as treatment-ineligible. This improvement was achieved with sensitivity from 51% or 52% increasing to 80%, while maintaining reasonable specificity (73%).

1.	Ratziu V, Charlotte F, Heurtier A, Gombert S, Giral P, Bruckert E, Grimaldi A, et al. Sampling variability of liver biopsy in nonalcoholic fatty liver disease. Gastroenterology 2005;128:1898-1906.

2.	Magrigal Pharmaceuticals. Madrigal Announces New Two-Year Data from the Compensated MASH Cirrhosis Arm of the MAESTRO-NAFLD-1 Trial Demonstrating Potential Benefit of Rezdiffra™ (resmetirom) in Patients with Compensated MASH Cirrhosis. In; 2025.
